# A second hotspot for pathogenic exon-skipping variants in *CDC45*

**DOI:** 10.1038/s41431-024-01583-1

**Published:** 2024-03-11

**Authors:** Kelly Schoch, Mischa S. G. Ruegg, Bridget J. Fellows, Joseph Cao, Sabine Uhrig, Stephanie Einsele-Scholz, Saskia Biskup, Samuel R. A. Hawarden, Vincenzo Salpietro, Valeria Capra, Undiagnosed Diseases Network, Chris M. Brown, Andrea Accogli, Vandana Shashi, Louise S. Bicknell

**Affiliations:** 1 Division of Medical Genetics, Department of Pediatrics, Duke University School of Medicine, Durham, NC USA; 2 Department of Biochemistry, University of Otago, Dunedin, New Zealand; 3 Division of Pediatric Radiology, Department of Radiology Duke University School of Medicine, Durham, NC USA; 4 Institute of Clinical Genetics, Klinikum Stuttgart, Stuttgart, Germany; 5 Center for Human Genetics Tuebingen and CeGaT GmbH, Tuebingen, Germany; 6 Department of Pathology, Dunedin School of Medicine, University of Otago, Dunedin, New Zealand; 7 Department of Biotechnological and Applied Clinical Sciences, University of L’Aquila, L’Aquila, Italy; 8 Genomics and Clinical Genetics, IRCCS Istituto Giannina Gaslini, Genoa, Italy; 9 Department of Specialized Medicine, Division of Medical Genetics, McGill University Health Centre, Montreal, QC Canada; 10 Department of Human Genetics, Faculty of Medicine, McGill University, Montreal, QC Canada

**Keywords:** Disease genetics, Medical genetics

## Abstract

Biallelic pathogenic variants in *CDC45* are associated with Meier-Gorlin syndrome with craniosynostosis (MGORS type 7), which also includes short stature and absent/hypoplastic patellae. Identified variants act through a hypomorphic loss of function mechanism, to reduce CDC45 activity and impact DNA replication initiation. In addition to missense and premature termination variants, several pathogenic synonymous variants have been identified, most of which cause increased exon skipping of exon 4, which encodes an essential part of the RecJ-orthologue’s DHH domain. Here we have identified a second cohort of families segregating *CDC45* variants, where patients have craniosynostosis and a reduction in height, alongside common facial dysmorphisms, including thin eyebrows, consistent with MGORS7. Skipping of exon 15 is a consequence of two different variants, including a shared synonymous variant that is enriched in individuals of East Asian ancestry, while other variants in trans are predicted to alter key intramolecular interactions in α/β domain II, or cause retention of an intron within the 3ʹUTR. Our cohort and functional data confirm exon skipping is a relatively common pathogenic mechanism in *CDC45*, and highlights the need for alternative splicing events, such as exon skipping, to be especially considered for variants initially predicted to be less likely to cause the phenotype, particularly synonymous variants.

## Introduction

CDC45 forms part of the CDC45-MCM-GINS (CMG) helicase, an 11-member complex which activates the MCM helicase activity for unwinding of the DNA, polymerase entry and the initiation of replication. The CMG helicase is active as part of the larger pre-initiation complex (preIC), during DNA replication initiation. The recruitment and activation of CDC45 is complex and governed by different protein-protein interactions within the preIC and signalling activity by CDK [1]. CDC45 is composed of two RecJ-like α/β domains, with an intermediate domain in between, connected by linker regions [2]. CDC45 sits to the outside of the MCM helicase ring, and contacts both MCM and GINS via multiple sites throughout the protein. The intricacies of this pathway are still being uncovered, such as the recent multiple descriptions reporting dynamic interactions linking DONSON, MCM helicase and GINS (and indirectly CDC45) as being necessary for preIC assembly and activity [3–5].

Meier-Gorlin syndrome (MGORS) is characterised by reduced growth, microtia and patella hypo/aplasia and is a disorder associated with components involved in the organisation and initiation of DNA replication [6]. Biallelic variants in *CDC45* are associated with a distinct spectrum of clinical features designated as MGORS7 (MIM 617063) [6–10]; while craniosynostosis is almost always present, many patients also have microtia and patella hypo/aplasia, more typical of MGORS. There does not appear to be a straightforward relationship between variant type and severity of presentation; this is exemplified by a sib-pair identified in the initial *CDC45*-cohort where one sibling has typical MGORS features in the absence of craniosynostosis, whereas the younger sibling has a clear craniosynostosis phenotype [7].

All variants in *CDC45* are predicted to act in a hypomorphic loss-of-function manner, with patient cells showing reduced *CDC45* transcript and protein levels [7]. Missense variants affect highly conserved residues across the protein. Within the large cohort initially identified, there was a cluster of variants around exon 4 [7]. Exon 4 is an in-frame exon that encodes an essential part of α/β domain I, which forms part of a domain orthologous to RecJ DHH. Interestingly, this exon shows low levels of alternative splicing in control cell lines. In the presence of patient variants, skipping of exon 4 is increased, reducing the overall amount of canonical *CDC45* transcript. Two of these variants are synonymous, indicating the importance of motifs within the exon in influencing exon skipping. Beyond exon 4, exon 7 (NM_001178010.2) also shows alternative splicing, with the canonical *CDC45* transcript lacking this exon (NM_003504.5). Altered splicing efficiencies of this exon through a pathogenic synonymous variant also reduce overall transcript levels, causing a phenotype typical for MGORS7 in two sibs [8].

We have collected a cohort of families with variants in *CDC45* that indicate a second hotspot cluster influencing exon skipping of exon 15 (NM_003504.5). We confirm the effects of these variants on canonical splicing using a minigene splicing assay, and provide evidence supporting pathogenicity for the second variant present in each family. Our findings confirm that exon skipping of in-frame exons is a relatively common pathogenic mechanism in *CDC45*. Synonymous variants that affect RNA biogenesis represent a significant class of pathogenicity and should be more strongly prioritised in variant filtering and curation of *CDC45*.

## Materials and methods

### Ethical approval

Ethical approval for this study was obtained from the Health and Disability Ethics Committee, New Zealand (16/STH/3). F1-1 was enrolled in a research study approved by the central IRB at the National Human Genome Research Institute (NHGRI, 15-HG-0130) and locally by the Duke University IRB (Pro00056651). All families agreed to participate in this study, and separate consent was obtained for the use of photographs.

### Minigene assay

*CDC45* exons 14–17 were PCR amplified from control genomic DNA using a forward primer located in intron 14 and a reverse primer located in intron 17, incorporating at least 20 bp of the intron and then cloned into the pSpliceExpress splicing vector [11]. Site directed mutagenesis was performed to incorporate either c.1416C>T or c.1440+1G>A, with all *CDC45* inserts in the plasmids confirmed by Sanger sequencing. Primer sequences are available on request. HEK293FT cells were transfected with the constructed plasmids using Lipofectamine-2000 (Invitrogen). After 24 h, RNA was extracted using Qiagen RNeasy Mini kit (Qiagen) and cDNA synthesized using SuperScript IV VILO Mastermix with ezDNase kit (Invitrogen). Plasmid-specific oligonucleotides (sense: CCTGCTCATCCTCTGGGAGC and anti-sense: AGGTCTGAAGGTCACGGGCC) were used in an RT-PCR using Phusion Flash II polymerase mastermix (Thermo) (denaturation 98 °C for 30 s, annealing 67 °C for 10 s, extension 72 °C for 1 min, for 21 cycles), and products were visualized by agarose gel electrophoresis. Gel bands were extracted and purified with GeneJet Gel Extraction kit (Thermo Fisher Scientific) and Sanger sequenced to confirm the splicing effect.

### Sequence alignments and protein modelling

Amino acid sequences from different species were aligned with Clustal Omega [12]. Orthologous CDC45 protein RefSeq IDs: *Homo sapiens* (*H. sapiens*; NP_003495.1), *Pan troglodytes* (*P. troglodytes*; XP_016795191.1), *Canis lupus familiaris* (*C. familiaris*; XP_543547.3), *Mus musculus* (*M. musculus*; NP_033992.2), *Gallus gallus* (*G. gallus*, XP_415070.3), *Danio rerio* (*D. rerio*; NP_998551.1). *Drosophila melanogaster* (*D. melanogaster*; NP_569880.1), *Anopheles gambiae* (*A. gambiae*; XP_320573.1), *Caenorhabditis elegans* (*C. elegans*; NP_497756.2), *Saccharomyces cerevisiae* (*S. cerevisiae*; NP_013204.1), and *Schizosaccharomyces pombe* (*S. pombe*; NP_594693.1). Jalview (v2.11.1.4; ELIXIR) [13] was used to annotate and colour protein alignment by percentage identity. Vertebrate *CDC45* genomic sequences were aligned manually for *H. sapiens* (NC_000022.11), *P. troglodytes* (NC_072420.1), *C. familiaris* (NC_051830.1), *M. musculus* (NC_000082.7), *Rattus norvegicus* (*R. norvegicus*; NC_051346.1). The protein structure of human CDC45 (PDB code: 5DGO) [14] was modelled in PyMOL (Version 1.2r3pre, Schrödinger, LLC).

## Results

As part of our ongoing studies into variants in *CDC45*, we collected a cohort of three families where diagnostic testing had identified variants in *CDC45*. All five affected individuals have each inherited one variant that potentially altered splicing around exon 15 (NM_003504.5) (Table 1). Clinical features of all five individuals are listed and compared to previous publications (Table 2, Supplementary Table 1) and pedigrees are displayed (Supplemental Fig. 1A).

**Table 1:** Genetic details of *CDC45* variants identified in this study.

Family	Variant 1/Paternally inherited variant				Variant 2/maternally inherited variant			
	Variant details	gnomAD v4		CADD score	Variant details	gnomAD v4		CADD score
		overall	popmax			overall	popmax	
F1-1*	c.204G>A, p.(Gln68 =)	absent	absent	26.2	c.1416C>T, p.(His472 =)	0.006%	0.14% (E Asian)	9.4
F2-1 – F2-3	c.*1+5G>A	0.0005%	0.002% (Admixed American)	24.7	c.1416C>T, p.(His472 =)	0.006%	0.14% (E Asian)	9.4
F3-1	c.290T>C (p.Val97Ala)	absent	absent	25.7	c.1440+1G>A	0.0003%	0.002% (E Asian)	34

RefSeq: NM_003504.5. *Parental genetic information is not available for Individual F1-1.

**Table 2:** Comparison of clinical features of individuals affected by MGORS7.

	F1-1	F2-1	F2-2	F2-3	F3-1	Total this paper	Li et al.	Previous cases total	Overall total
Birth									
Short stature	na	–	+	na	+	2/3	na	3/9	5/12
Microcephaly	na	–	+	+	–	2/4	+	8/11	11/16
Low birth weight	na	–	+	na	+	2/3	na	8/17	10/20
Most recent exam									
Short stature	+	+	+	na	+	4/4	na	12/15	16/19
Microcephaly	+	+	+	na	+	4/4	na	15/15	19/19
Low weight	+	+	+	na	+	4/4	na	11/14	15/18
MGORS characteristics									
Microtia	+	+	+	+	+	5/5	+	16/17	21/22
Absent/small patella	+	na	na	na	–	1/2	na	8/11	9/13
*CDC45-*related characteristics									
Craniosynostosis	+	–	+	+	+	4/5	+	17/18	21/23
Ano-rectal malformation	–	+	+	na	+	3/5	na	8/16	11/21
Thin eyebrows	+	na	na	na	–	1/2	na	18/18	19/20
Cardiac defects	–	+	+	+	+	3/5	-	7/16	10/21
Developmental delay	+	+	na	na	+	3/3	na	6/15	9/18

Li et al. is listed separately since this case has also inherited the c.1416C>T variant. na, not available.

In family 1, a 9-year-old boy of Chinese ancestry presented with severe growth delay (height −3.87 standard deviations (SD), occipitofrontal circumference (OFC) −5.43 SD, weight −7.19 SD) and craniosynostosis. Individual F1-1 has facial asymmetry, with a flat midface, short palpebral fissures, thin upper lip and very thin eyebrows, which is a common feature in individuals with *CDC45* variants (Supplementary Figure BA). Both patellae are absent, and of note, he has hypoplastic thumbs and great toes (Supplementary Fig. 1C–E) (Supplementary Table 1). Ears are small, cupped, low-set and posteriorly rotated. He also has mild developmental delay, moderate mixed hearing loss, vertebral fusions, and pancreatic insufficiency. Following genome sequencing, Individual F1-1 was found to be heterozygous for c.204G>A, p.(Gln68 =) (NM_003504.5), which lies at the terminal base of exon 3 and so is within the essential splice donor site. While a phenotype-agnostic approach was employed for variant filtering, this variant was flagged because a similar variant has been reported previously (c.203A>G, p.Gln68Arg) in a sib-pair, which caused MGORS7 with discordancy for craniosynostosis [7]. Parent samples were not available to filter for variants in trans, however manual inspection suggested the synonymous variant c.1416C>T, p.(His472 =) (NM_003504.5) as a candidate. It was the only other variant within the coding sequence and synonymous variants in *CDC45* have been reported as previously to be pathogenic [7, 8]. During the functional investigation of this variant, a case report was published describing this variant as a VUS, in trans with a pathogenic variant, in a foetus with features of MGORS7; however no functional studies for the c.1416C>T variant were performed [9].

There have been three affected cases in Family 2. Individual F2-1 presented with low growth parameters (height −2.41 SD, OFC −3.04 SD, weight −3.4 SD). Development was mildly delayed, especially motor function. Individual F2-1 shows midface hypoplasia, a curved nose with downturned nasal tip, a full lower lip and bilateral microtia, but no craniosynostosis. She has cardiac abnormalities and anterior anal atresia. Individual F2-2 showed *in utero* growth retardation, with reduced length and microcephaly at birth. Facial dysmorphism included midface hypoplasia, hypertelorism, a full lower lip and low-set, slightly dysplastic ears. Additionally, Individual F2-2 had cardiac abnormalities and anal atresia with a perianal fistula. Surgery was performed to correct his bilateral coronal synostosis, however following this surgery, he died from a pulmonary embolism. For Individual F2-3, developmental abnormalities were detected by ultrasound during pregnancy, including microcephaly, suggestive craniosynostosis and cardiac anomalies. Given the family history, the decision was made to terminate the pregnancy. Exome sequencing of Individual F2-2 and both parents was undertaken, and in considering the phenotype, two variants in *CDC45* were identified in a compound heterozygous manner: c.1416C>T, p.(His472 =) (NM_003504.5), the same variant observed in Individual F1-1, and c.*1+5G>A (NM_003504.5), an intronic variant close to the splice donor site of the last coding exon (exon 18), where the terminal intron interrupts the 3′UTR sequence of *CDC45*. Additional exome sequencing of Individuals F2-1 and F2-3 also identified both variants in a compound heterozygous manner.

Individual F3-1 was born at 38 weeks gestation, with reduced length (−3.99 SD) and weight (−2.28 SD). At the most recent examination, he showed primordial dwarfism, with height and OFC both more severe than −4 SD (Supplementary Fig. 1F). Individual F3-1 demonstrated global developmental delay and had right plagiocephaly (Supplementary Fig. 1G). Individual F3-1 showed facial dysmorphism including frontal bossing, downslanting palpebral fissures with asymmetric eyes, micrognathia and low-set, small protruding ears (Supplementary Fig. 1H). Individual F3-1 has skeletal abnormalities including vertebral segmentation defects, but patellae have developed normally. Congenital heart defects were also present, along with an ano-rectal malformation and fistula. Research based trio exome sequencing analysing a variety of inheritance models indicated compound heterozygosity for variants in *CDC45*: a missense variant (c.290T>C (p.Val97Ala) (NM_003504.5)), and an intronic variant affecting the essential splice donor site of exon 15 (c.1440+1G>A) (NM_003504.5).

In two families (F1, F2) there was a shared synonymous variant in exon 15, c.1416C>T. This variant is rare in control groups and appears to be enriched in individuals of East Asian descent (gnomAD v4 allele frequency = 0.001449). Prediction tools such as SpliceAI or MaxEntScan did not predict an alteration in splicing, but visual comparison of exonic splicing enhancer and silencer motifs (Alamut Visual Plus software) predicted the creation of a SRp55 motif when the variant is present (Supplemental Fig. 2). SRp55 is an established splicing factor, with roles in alternative splicing [15, 16], although the functional consequence of a predicted single extra motif is not clear. However, given this prediction, and that this recurrent variant has been inherited in trans with different strong candidate variants, we investigated this synonymous variant further.

**Fig. 1:**
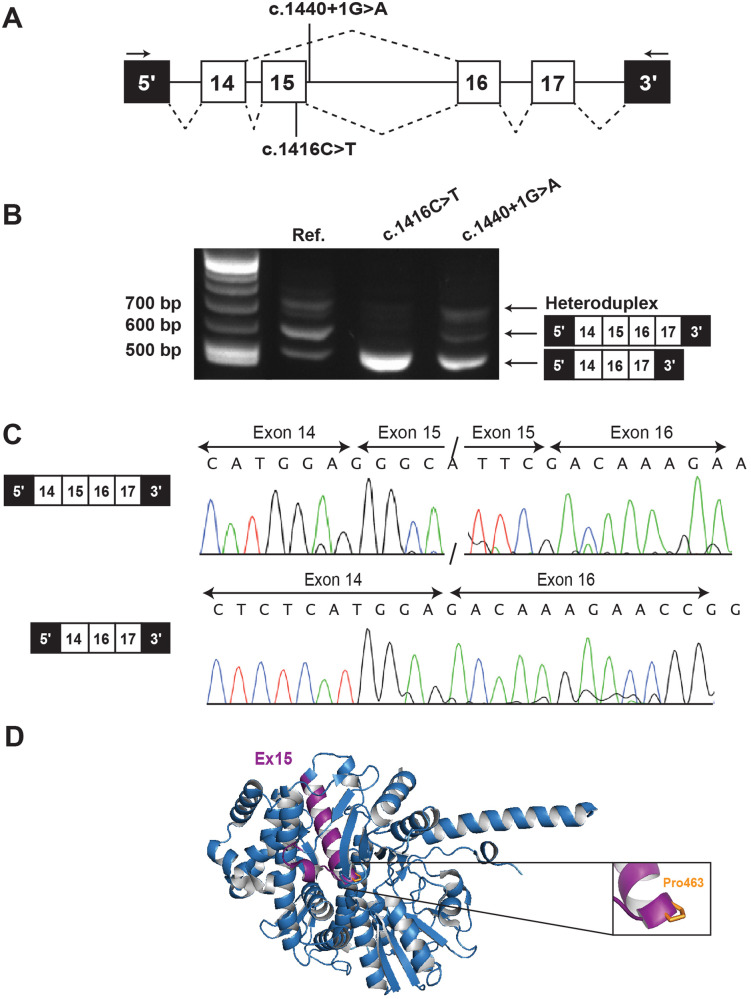
Variants clustering to *CDC45* exon 15 cause in-frame exon skipping. **A** Schematic of the *CDC45* gene region amplified in minigene splicing assay, showing position of two variants under study with the location of RT-PCR primers noted as arrows. **B** RT-PCR of *CDC45* splicing products from plasmid-derived mRNA transiently expressed in HEK293FT cells, with two major transcript products produced. Heteroduplex bands are visible as faint high molecular weight products in all lanes. **C** Sanger sequencing confirms the higher molecular weight product (~580 bp) in (**B**). represents the canonical transcript for this region of *CDC45*, whereas the lower molecular weight product (~450 bp) reflects a novel transcript skipping exon 15. **D**. Protein model of CDC45 (PDB code: 5DGO). The region of CDC45 encoded by exon 15 (purple) is within α/β domain II of CDC45 and includes a core α-helix, so absence of this α-helix is expected to impact protein stability. The location of Pro463, substituted in a previously established pathogenic missense variant, is noted within the same α-helix.

To test the potential effect of this shared synonymous variant (c.1416C>T) on transcript splicing, as well as the effect of the c.1440+1G>A variant in F3-1, which has not been studied before, we undertook a minigene splicing assay using a pSpliceExpress plasmid [11]. Genomic regions from *CDC45* exons 14–17, plus flanking intronic sequences, were introduced into the pSpliceExpress plasmid, and a variant plasmid was synthesized using site-directed mutagenesis to introduce either the c.1416C>T or the c.1440+1G>A variant (Fig. 1A). Following transfection in HEK293FT reporter cells and RNA extraction, a RT-PCR was undertaken to identify transcripts produced from this part of the *CDC45* gene (Fig. 1B). Two strong products were identified across the different minigene plasmids tested and bands were excised from the gel and sequenced to confirm identity (Fig. 1C). The reference transcript produced a predominant product consisting of the canonical transcript of exons 14–17, and a smaller RT-PCR product that sequencing confirmed was exons 14–17 but skipping exon 15. The synonymous variant (c.1416C>T), which lies in exon 15, caused almost complete skipping of exon 15. The second variant (c.1440+1G>A), which lies in the essential splice donor site of exon 15, also generated the same new product in which exon 15 is skipped, however this product was not as frequently spliced into a transcript, and more of the canonical transcript was present. Exon 15 is an in-frame exon encoding 28 amino acids and so CDC45 protein is still predicted to be produced. Exon 15 translates to a core α-helix within CDC45 α/β domain II. This helix structure also contains the residue affected by the shared p.Pro463Leu substitution that was identified in three South Asian individuals from the first cohort (P4, P5, P6) [7], where it was predicted to have severe effects on helix and overall protein stability. Although loss of exon 15 would still produce a protein, it would likely have severe effects on structure and function.

**Fig. 2:**
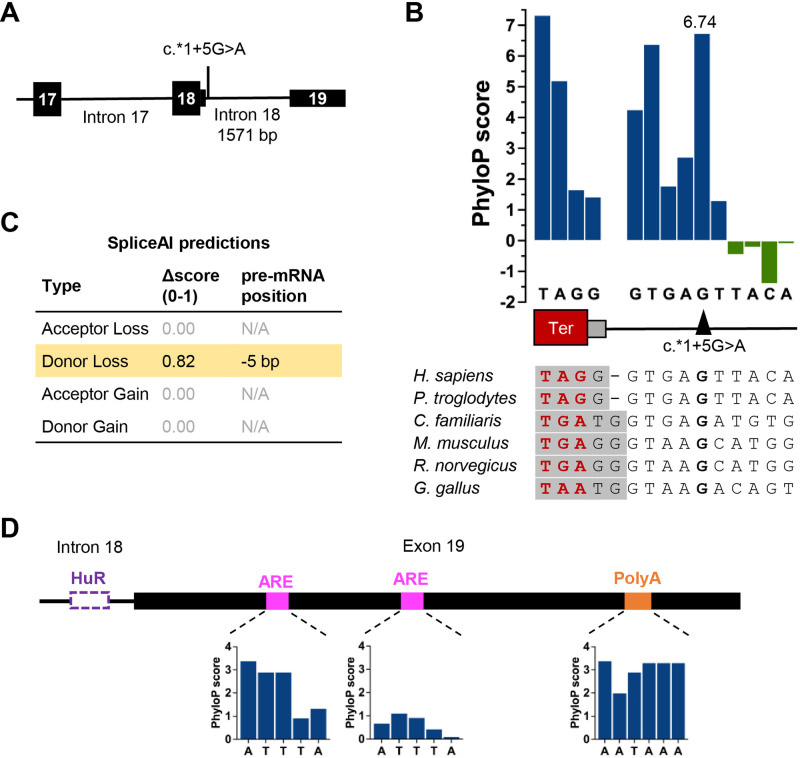
A splicing variant in the terminal intron is expected to alter the 3′UTR sequence of CDC45. **A** Schematic of 3′ region of the *CDC45* gene showing the position of c.*1+5G>A in the terminal intron (intron 18, NM_003504.5). **B** Nucleotide conservation and PhyloP score at the exon/intron boundary, with the site of the identified intronic variant indicated. Ter, termination codon (red). Grey box, first 1–2 nucleotides of the 3ʹUTR. PhyloP analysis measured nucleotide evolutionary conservation across 100 vertebrate species from the UCSC genome database. PhyloP scores represent -log p values under a null hypothesis of neutral evolution. Positive scores (blue) are predicted to be conserved; negative values (green) predict faster-evolving nucleotides. The +5 position is the most strongly conserved intronic position, with a phyloP score of 6.74. **C** Output scores from SpliceAI, strongly supporting loss of the exon 18 splice donor site (0.8 is the threshold for a high precision prediction). No cryptic splice donor site is predicted, suggesting the entire intron could be retained and form part of the 3ʹUTR sequence. **D** Schematic of predicted motifs within the canonical human *CDC45* 3ʹUTR, with PhyloP scores of ARE and polyA sites. The intronic predicted HuR site in the human intron is also indicated (purple).

In addition to the synonymous variant c.1416C>T, cases from family F2 were compound heterozygous for the intronic variant c.*1 + 5G>A. Interestingly, *CDC45* intron 18 separates two exons that encode the 3ʹUTR, with the exon 18 splice donor site one base downstream of the termination codon (Fig. 2A). The presence of this intron is conserved through most vertebrates, albeit with some small variation in having one or two 3ʹUTR nucleotides present upstream of the splice donor site (Fig. 2B). The guanine positioned 5 bases into the intron is highly conserved through 100 vertebrates, and in fact has the highest PhyloP [17] score amongst intronic nucleotides in this region (Fig. 2B), suggesting a substitution at this position would be deleterious to the exon 18 splice donor site. SpliceAI [18] scores agree with this hypothesis, with a strong prediction to impact splicing at the exon 18 splice donor site (SpliceAI Δ score donor loss = 0.82), likely due to strong conservation of this guanine in interaction with the U1 spliceosome [19]. No gain of a donor or acceptor site was predicted (even when the sequence analysed was extended to include all of intron 18 and exon 19 sequences) (Fig. 2C). Taken together these would predict the variation would cause retention of this final intron in the *CDC45* transcript. The consequence would be a significant lengthening of the 3ʹ UTR sequence by an additional 1571 nucleotides. In the canonical short (132 nucleotide) 3ʹUTR we predicted two AU-Rich Elements (AREs) that could selectively stabilise or destabilise the mRNA and the conserved polyadenylation signal (AAUAAA) [20, 21], indicating that this 3ʹUTR has multiple conserved regulatory motifs to control mRNA metabolism (Fig. 2D). In the longer UTR, no additional ARE, conditional destabilising elements or PolyA signals were predicted [22, 23], however a U rich element that could represent HuR RNA binding sites were predicted by BRIO (Fig. 2D) [24]. We speculate the longer 3ʹUTR may disrupt the predicted regulatory elements in the short 3’UTR, or additional motifs could bind RNA-binding proteins or miRNAs to destabilise the RNA, as has been shown with other human UTRs [25, 26].

**Fig. 3:**
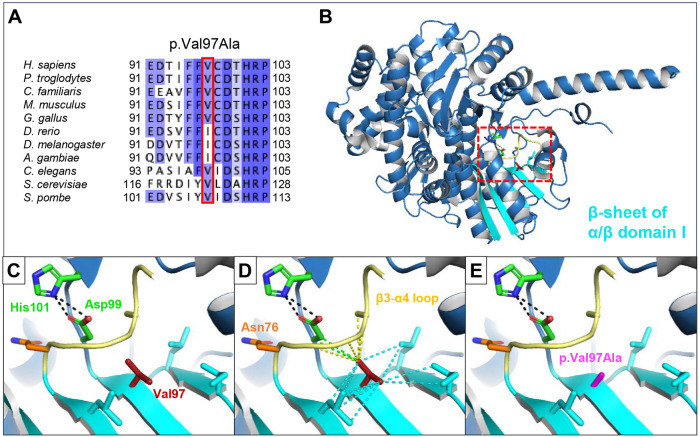
Structurally modelling the effect of the CDC45 p.Val97Ala substitution. **A** Clustal Omega alignment of eukaryotic CDC45 protein sequence coloured by percentage identity shows Val97 is well conserved through to fungi. **B** Val97 is located in the protein core and within the parallel β-sheet (cyan) of α/β domain II. **C** Zoomed in image of the red box highlighted in B. showing wildtype Val97 sidechain (red sticks) is positioned in a hydrophobic pocket near conserved Asp99 and His101 residues (green sticks), corresponding to the catalytic residues in RecJ orthologues. Asp-His hydrogen bonds are represented as black dashes. **D** Wildtype Val97 sidechain forms stabilising hydrophobic contacts (dashes) within the β-sheet secondary structure (cyan), as well as to Asp99 (green), and to the β3-α4 loop (yellow) which harbors Asp76, the residue affected by a previously established pathogenic variant. **E** Mutagenesis modelling of the p.Val97Ala substitution (magenta) causes the loss of the Val97 sidechain specific hydrophobic contacts shown in D.

In addition to the splice-altering variant, Individual F3-1 is heterozygous for a missense variant, c.290T>C, p.(Val97Ala). The substitution affects a conserved valine residue located within the parallel β-sheet of α/β domain I (Fig. 3A, B). This β-sheet forms the known contact site between CDC45 and GINS1 within the CMG complex [2]. Val97 is positioned closely upstream of the conserved Asp99 and His101 residues, which are homologous to the Asp-His residue pair that is involved in catalysis in RecJ and other DHH phosphoesterase superfamily proteins [27] (Fig. 3C). The Val97 sidechain is buried inside the hydrophobic core and forms a network of hydrophobic contacts which includes extensive interactions with the β3-α4 loop, likely stabilising it within the core (Fig. 3D). Notably, the β3-α4 loop contains Asp76, the residue affected by the p.Asn76His substitution identified in an individual with bicoronal synostosis [7]. The Val97Ala substitution would reduce the hydrophobicity at this conserved site, weakening the hydrophobic interactions and likely perturbing protein stability (Fig. 3E).

## Discussion

Here we describe the identification of clinical and molecular findings in five individuals with features of MGORS7. We identify a second hotspot for synonymous variants causing exon skipping in *CDC45*, highlighting the importance of such variants, which can be overlooked as causing Mendelian disorders [28–30].

The five individuals from three families reported here had biallelic variants in *CDC45*: all with one synonymous and/or splicing variant shown to cause skipping of exon 15 in trans with a pathogenic variant (presumed in trans for Individual F1-1 since parents are unavailable). Where the relevant information was available, short stature, microcephaly, and microtia and developmental delays were seen in all individuals, and 4/5 had craniosynostosis. Absent/hypoplastic patellae, a distinctive feature of MGORS7, was not seen in at least one individual in this cohort. Ano-rectal malformations and cardiac defects were observed but more variably [6, 7]. Interestingly, two individuals in this cohort had pancreatic insufficiency and thus perhaps this feature may be part of the MGORS7 spectrum. Overall, the majority of the five individuals had the salient MGORS7 features of short stature, microtia and absent/hypoplastic patellae; variability in manifestations as seen in these individuals is consistent with prior reports of MGORS7 [7].

In the initial cohort previously described, exon 4 was sensitive to alternative splicing by either splice-site variants or synonymous variants affecting exon skipping motifs [7]. Exon 4 is an in-frame exon and encodes a conserved region of the α/β domain I. Alternative splicing has been observed in mRNA from control individuals [7], and exon 3–5 junction sequencing data is present in low levels in several tissues in GTEx mRNA data, including in both established cell line models in GTEx. Given the conserved amino acids encoded by exon 4, the toleration of this alternative splicing is unclear. It would be interesting to examine developmentally-focused sequencing data for the presence of this exon skipping event to further analyse whether this is simply a byproduct of sequence motifs, or whether this alternatively spliced isoform may have a biological role.

With the cluster of variants described here, we propose that exon 15 represents another exon sensitive to exon skipping. Exon 15 is also in-frame and encodes a core α-helix that is likely required for overall stability of the C-terminal DHHA1 domain. While this exon skipping was also detected in low levels in the reference allele in our minigene splicing assay, skipping of exon 15 has not been observed in GTEx datasets. This could reflect a higher sensitivity for exon skipping motifs in our reporter cell model (HEK293FT). Minigene splicing assays are a valuable tool for studying candidate splicing variants but do have limitations in relating results from reporter models to tissues relevant to the disorder [31]. Regardless, the significant proportion of exon skipping product versus canonical transcript is strongly supportive of the pathogenic effects of both variants identified here in reducing canonical CDC45 transcript levels. Such a decrease likely confers a hypomorphic loss-of-function consequence at the protein level.

The synonymous variant studied (c.1416C>T) was found in two individuals in this cohort, both of East Asian ancestry. The same variant has also been reported as a VUS in a Chinese family who had a foetus with a prenatal diagnosis of MGORS7 [9]. This variant is present in gnomAD, and while still rare, is significantly more common in the East Asian group and shows an enrichment in individuals of South Asian ancestry. This population enrichment has diagnostic implications, and we recommend it should be a priority variant to be considered in individuals of East and South Asian ancestry who present with clinical features similar to MGORS7 and/or craniosynostosis. We note in ClinVar that this variant has been classified as benign (SCV002504460.2) or VUS (SCV003490501.1) by other groups, although no supporting evidence was provided for the benign classification. We recommend a fresh assessment of this variant, given the functional evidence we have presented here – we have classified this variant as Likely Pathogenic (SCV001249395.18).

In addition to alternative splicing, synonymous variants can affect transcription, mRNA stability and protein folding kinetics (if the codon is mutated to a rare codon) [32–34]. While the potential deleterious effects of synonymous variants have long been recognised [35, 36], the interpretation and confirmation of deleterious effects of such variants is likely harder to achieve within a diagnostic environment, where the ability to follow up candidate variants using functional experiments is likely reduced. Despite this, we propose that considering synonymous variants in a recessive disorder should be prioritised, especially if there is other supportive evidence, such as characteristic clinical features. If the variant is homozygous, then consider whether identity-by-descent could have occurred via an ancestral locus (particularly if a consanguineous union) which could indicate an increased likelihood of pathogenicity. in considering a compound heterozygous inheritance model, if there is a clear pathogenic variant in trans, then the PM3 criterion from the AMP/ACMG guidelines can be applied [37], potentially helping to reclassify this variant. Notably, both variants in Individual F1-1 were synonymous, with only the c.204G>A flagged by splice predictor computational tools given its location within the splice donor region of exon 3. The c.1416C>T variant was universally predicted to be benign, with low prediction scores (SpliceAI score = 0.01, CADD = 9.43); indeed the clinical laboratory that initially performed the exome sequencing on Individual F1-1 did not report the c.1416C>T variant, citing in part the ClinVar reports for this variant.

Similarly, we detected a variant, c.*1+5G>A, predicted to cause intron retention and therefore a longer 3’UTR, likely causing disruption and destabilisation of the transcript by nonsense mediated decay and other mechanisms [23]. Variants affecting UTR regions represent another class of variant in which interpretation is difficult. The +5 guanine in an intron is highly conserved as it is required for recognition by the U1 spliceosome, and substitution of this nucleotide is strongly predicted to impair correct splicing of the final intron.

We have demonstrated that synonymous variants are a common mechanism of pathogenicity for variants in *CDC45*. Synonymous variants are more difficult to filter and prioritise during computational analysis in genetic testing protocols, and some pipelines may even exclude them from the final filtered candidate list. Phenotypes associated with variants in *CDC45* are inherited in a recessive manner, and for all previous individuals in whom we identified a new synonymous candidate variant, the patient had a clearly pathogenic variant, often a frameshift, in trans [7, 8]. This provided confidence that *CDC45* was likely the correct gene diagnosis and supported a more thorough examination of other variants present, aided by functional investigation. However, in this report, both variants were synonymous in one of the individuals while in another family, the synonymous variant was in trans with a 3ʹUTR variant, serving as a reminder of the potential pathogenicity of synonymous/non-coding variants, and the benefit of retaining such variants in an analytical pipeline. Given the significant increase in the level of transcriptomic variation driven by technological advances [38], one prioritisation approach could be to filter variants based on proximity to exons sensitive to alternative splicing. While splicing prediction has significantly improved, with tools such as spliceAI [18], exon skipping remains difficult to predict and is an area for further development, likely guided by machine learning approaches.

In summary, our report provides expanded clinical and genetic information on MGORS7 due to biallelic variants in *CDC45*. We highlight the importance of exon skipping as a disease mechanism in this disorder, thereby emphasizing that synonymous variants in this gene can be disease associated.

### Supplementary information

Supplementary Table 1

Supplemental Figures

Supplemental Information - UDN Member List

## Data Availability

Genetic data is not available in order to protect individuals’ genetic privacy.
