# Supplementary material for: A second hotspot for pathogenic exon-skipping variants in CDC45
Source: Eur J Hum Genet. 2024 Mar 11;32(7):786–94. doi: 10.1038/s41431-024-01583-1 (PMC11219862; doi:10.1038/s41431-024-01583-1)
Supplement: Supplementary file 2 — Supplemental Figures [file 41431_2024_1583_MOESM2_ESM.pdf]

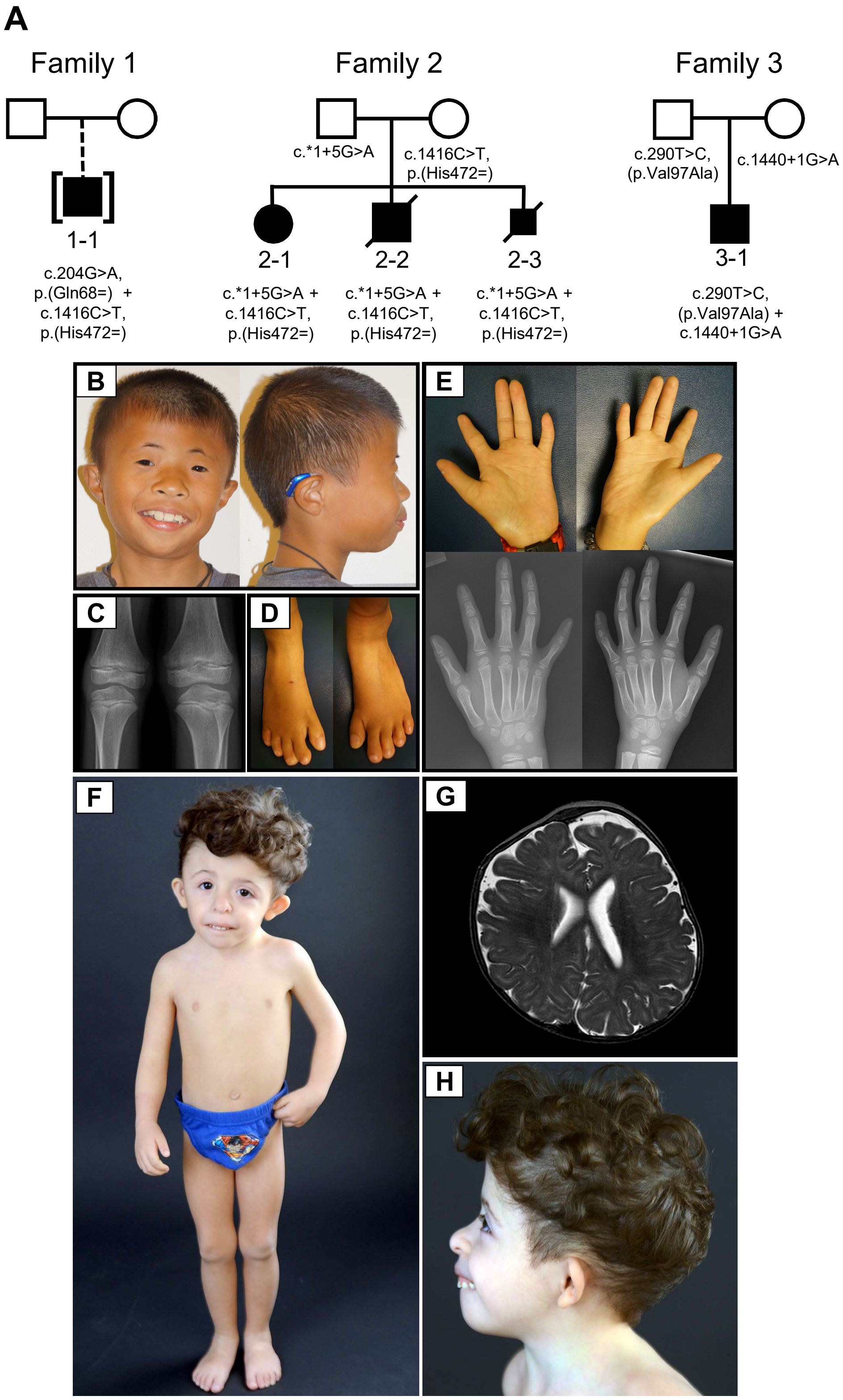

Supplemental Figure 1. Pedigrees and clinical features of individuals with variants in *CDC45*.

A. Pedigrees for each of the families involved in the study. B-E. Clinical features of Individual F1-1, showing B, sparse eyebrows, short palpebral fissures, facial asymmetry and a thin upper lip; C, congenital bilateral patellar aplasia; D, hypoplastic great toes, E, radiographs demonstrating hypoplasia of the 1st metacarpal, 2nd and 5th digit middle phalanges with corresponding digit shortening on clinical exam. F-H. Clinical features of Individual F3-1, showing F, right plagiocephaly, facial features of downslanting palpebral fissures with asymmetric eyes, deviated nose, broad philtrum, micrognathia, and pointed chin; G, asymmetric right anterior and posterior synostotic plagiocephaly; H, small low-set ears, frontal bossing and protruding superior dental arch.

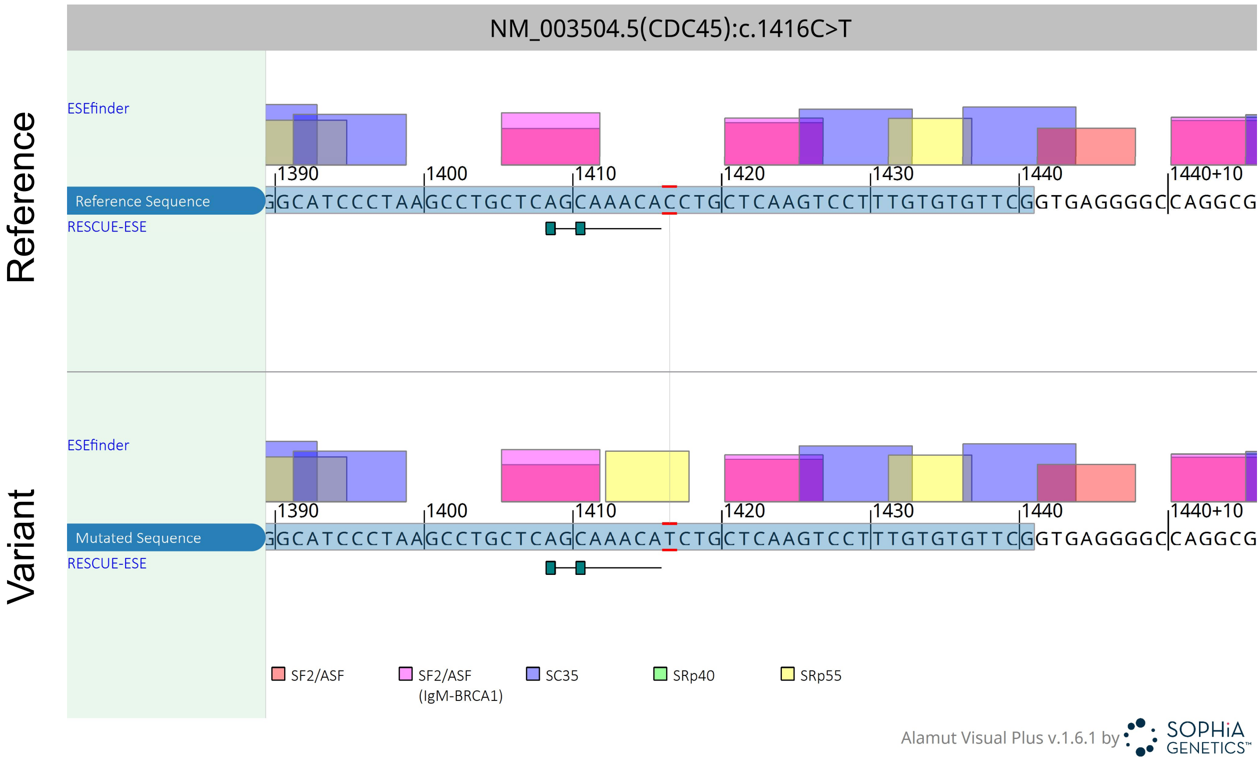

Supplemental Figure 2. Splice enhancer and silencer motif prediction for the c.1416C>T variant. Alamut Visual Plus (Sophia Genetics) prediction of splice enhancer and silencer motifs. The top half of the figure shows prediction with the reference allele and the bottom half indicates predictions when the variant is present (variant position indicated by red lines). An additional SRp55 splice enhancer motif is predicted when the variant is present.
