# Supplementary material for: A second hotspot for pathogenic exon-skipping variants in CDC45
Source: Eur J Hum Genet. 2024 Mar 11;32(7):786–94. doi: 10.1038/s41431-024-01583-1 (PMC11219862; doi:10.1038/s41431-024-01583-1)
Supplement: Supplementary file 3 — Supplemental Information - UDN Member List [file 41431_2024_1583_MOESM3_ESM.docx]

**Members of the Undiagnosed Diseases Network**

Maria T. Acosta

David R. Adams

Ben Afzali

Aimee Allworth

Raquel L. Alvarez

Justin Alvey

Ashley Andrews

Euan A. Ashley

Carlos A. Bacino

Guney Bademci

Ashok Balasubramanyam

Dustin Baldridge

Jim Bale

Michael Bamshad

Deborah Barbouth

Pinar Bayrak-Toydemir

Anita Beck

Alan H. Beggs

Edward Behrens

Gill Bejerano

Hugo J. Bellen

Jimmy Bennett

Jonathan A. Bernstein

Gerard T. Berry

Anna Bican

Stephanie Bivona

Elizabeth Blue

John Bohnsack

Devon Bonner

Nicholas Borja

Lorenzo Botto

Lauren C. Briere

Elizabeth A. Burke

Lindsay C. Burrage

Manish J. Butte

Peter Byers

William E. Byrd

John Carey

Thomas Cassini

Sirisak Chanprasert

Hsiao-Tuan Chao

Ivan Chinn

Gary D. Clark

Terra R. Coakley

Laurel A. Cobban

Joy D. Cogan

Matthew Coggins

F. Sessions Cole

Brian Corner

Rosario Corona

William J. Craigen

Andrew B. Crouse

Vishnu Cuddapah

Michael Cunningham

Precilla D’Souza

Hongzheng Dai

Surendra Dasari

Joie Davis

Margaret Delgado

Esteban C. Dell'Angelica

Katrina Dipple

Daniel Doherty

Naghmeh Dorrani

Jessica Douglas

Emilie D. Douine

Dawn Earl

Lisa T. Emrick

Christine M. Eng

Kimberly Ezell

Elizabeth L. Fieg

Paul G. Fisher

Brent L. Fogel

Jiayu Fu

William A. Gahl

Rebecca Ganetzky

Emily Glanton

Ian Glass

Page C. Goddard

Joanna M. Gonzalez

Andrea Gropman

Meghan C. Halley

Rizwan Hamid

Neal Hanchard

Kelly Hassey

Nichole Hayes

Frances High

Anne Hing

Fuki M. Hisama

Ingrid A. Holm

Jason Hom

Martha Horike-Pyne

Alden Huang

Yan Huang

Wendy Introne

Gail P. Jarvik

Jeffrey Jarvik

Suman Jayadev

Orpa Jean-Marie

Vaidehi Jobanputra

Emerald Kaitryn

Oguz Kanca

Shamika Ketkar

Dana Kiley

Gonench Kilich

Shilpa N. Kobren

Isaac S. Kohane

Jennefer N. Kohler

Susan Korrick

Deborah Krakow

Elijah Kravets

Seema R. Lalani

Christina Lam

Brendan C. Lanpher

Ian R. Lanza

Kumarie Latchman

Kimberly LeBlanc

Brendan H. Lee

Richard A. Lewis

Pengfei Liu

Nicola Longo

Sandra K. Loo

Joseph Loscalzo

Richard L. Maas

Ellen F. Macnamara

Calum A. MacRae

Valerie V. Maduro

AudreyStephannie Maghiro

Rachel Mahoney

May Christine V. Malicdan

Rong Mao

Ronit Marom

Gabor Marth

Beth A. Martin

Martin G. Martin

Julian A. Martínez-Agosto

Shruti Marwaha

Allyn McConkie-Rosell

Alexa T. McCray

Elisabeth McGee

Matthew Might

Mohamad Mikati

Danny Miller

Ghayda Mirzaa

Eva Morava

Paolo Moretti

Marie Morimoto

John J. Mulvihill

Mariko Nakano-Okuno

Stanley F. Nelson

Serena Neumann

Shirley Nieves-Rodriguez

Donna Novacic

Devin Oglesbee

James P. Orengo

Laura Pace

Stephen Pak

J. Carl Pallais

Jeanette C. Papp

Neil H. Parker

LéShon Peart

Leoyklang Petcharet

John A. Phillips III

Jennifer E. Posey

Lorraine Potocki

Barbara N. Pusey Swerdzewski

Aaron Quinlan

Ramakrishnan Rajagopalan

Deepak A. Rao

Anna Raper

Wendy Raskind

Adriana Rebelo

Genecee Renteria

Chloe M. Reuter

Lynette Rives

Amy K. Robertson

Lance H. Rodan

Jill A. Rosenfeld

Elizabeth Rosenthal

Francis Rossignol

Maura Ruzhnikov

Marla Sabaii

Jacinda B. Sampson

Timothy Schedl

Kelly Schoch

Daryl A. Scott

Elaine Seto

Vandana Shashi

Emily Shelkowitz

Sam Sheppeard

Jimann Shin

Edwin K. Silverman

Janet S. Sinsheimer

Kathy Sisco

Cara Skraban

Carson A. Smith

Kevin S. Smith

Lilianna Solnica-Krezel

Ben Solomon

Rebecca C. Spillmann

Andrew Stergachis

Joan M. Stoler

Kathleen Sullivan

Shirley Sutton

David A. Sweetser

Virginia Sybert

Holly K. Tabor

Queenie K.-G. Tan

Amelia L. M. Tan

Arjun Tarakad

Herman Taylor

Mustafa Tekin

Willa Thorson

Cynthia J. Tifft

Camilo Toro

Alyssa A. Tran

Rachel A. Ungar

Adeline Vanderver

Matt Velinder

Dave Viskochil

Tiphanie P. Vogel

Colleen E. Wahl

Melissa Walker

Nicole M. Walley

Jennifer Wambach

Jijun Wan

Michael F. Wangler

Patricia A. Ward

Daniel Wegner

Monika Weisz Hubshman

Mark Wener

Tara Wenger

Monte Westerfield

Matthew T. Wheeler

Jordan Whitlock

Lynne A. Wolfe

Heidi Wood

Kim Worley

Shinya Yamamoto

Zhe Zhang

Stephan Zuchner
